# Prediction and analysis of the modular structure of cytochrome P450 monooxygenases

**DOI:** 10.1186/1472-6807-10-34

**Published:** 2010-10-15

**Authors:** Demet Sirim, Michael Widmann, Florian Wagner, Jürgen Pleiss

**Affiliations:** 1 Institute of Technical Biochemistry, University of Stuttgart, Allmandring 31, 70569 Stuttgart, Germany

## Abstract

**Background:**

Cytochrome P450 monooxygenases (CYPs) form a vast and diverse family of highly variable sequences. They catalyze a wide variety of oxidative reactions and are therefore of great relevance in drug development and biotechnological applications. Despite their differences in sequence and substrate specificity, the structures of CYPs are highly similar. Although being in research focus for years, factors mediating selectivity and activity remain vague.

**Description:**

This systematic comparison of CYPs based on the Cytochrome P450 Engineering Database (*CYPED*) involved sequence and structure analysis of more than 8000 sequences. 31 structures have been applied to generate a reliable structure-based HMM profile in order to predict structurally conserved regions. Therefore, it was possible to automatically transfer these modules on CYP sequences without any secondary structure information, to analyze substrate interacting residues and to compare interaction sites with redox partners.

**Conclusions:**

Functionally relevant structural sites of CYPs were predicted. Regions involved in substrate binding were analyzed in all sequences among the *CYPED*. For all CYPs that require a reductase, two reductase interaction sites were identified and classified according to their length. The newly gained insights promise an improvement of engineered enzyme properties for potential biotechnological application. The annotated sequences are accessible on the current version of the *CYPED*. The prediction tool can be applied to any CYP sequence via the web interface at http://www.cyped.uni-stuttgart.de/cgi-bin/strpred/dosecpred.pl.

## Background

Cytochrome P450 monooxygenases (CYPs) are a ubiquitous protein family, existing in all eukaryotes, most prokaryotes and Archae. These heme-containing enzymes catalyze the monooxygenation of a large variety of substrates [1]. CYPs have an essential function in drug metabolism, hence focussed in the pharmaceutical industry [2]. Besides, they are of great interest for synthetical application in biotechnology as versatile biocatalysts [3]. A profound knowledge in the factors mediating selectivity and activity of these proteins is a prerequisite in the development of CYPs with improved properties. Therefore, deeper insights in the relationships between sequence, structure and function are of great interest.

According to Nelson's classification [4] CYPs are grouped into homologous families and superfamilies, predominantly based on sequence similarity. The sequence identity between proteins from different superfamilies is extremely low and may be less than 20% [5]. Only three amino acids are totally conserved, the glutamic acid and the arginine of the ExxR-motif, which is involved in stabilizing the core and heme-binding [6], and the heme-binding cysteine. However, the increasing number of crystal structures shows that despite this unusual variability the overall structure is highly conserved: CYPs consists of structural conserved modules that are essential for structure and function, and of variable regions that mediate the individual biochemical properties. The defined conserved secondary structures are named αA-L and β1-5 and could be identified in all CYP structures and make up the so called CYP-fold [7-9].

Most CYPs require interaction with a reductase to provide electrons, either as separate proteins or as fusion proteins. Depending on the nature of their electron transfer partner, CYPs are assigned to different classes. Although, no consensus has been reached in the definition of this classification, there are several proposed schemes which subdivide CYPs in up to nine classes [10-12]. The most general one, which was applied in this work, discriminates between two major classes of CYPs [13]: class I, which comprises mitochondrial and bacterial CYPs and class II which comprises CYPs interacting with a cytochrome P450 reductase-type (CPR-type) FMN/FAD reductase and represents a simplification of the widely accepted classification scheme by Kelly et al. in [1]. Further, there are CYPs known which do not need a reductase for their reaction [14]. Fusion proteins, such as the self-sufficient class II CYP 102A1 from *Bacillus megaterium *(P450 BM-3) which contains a heme domain and a reductase, as well as those CYPs which do not require any reductase interaction appear very rarely in nature [15]. Therefore, in most CYPs the interaction with their appropriate redox partner is prerequisite for their reaction to occur. Many different CYP isoenzymes interact with only one reductase, and it is assumed that CYPs of the same class are comparable in regard to their reductase interaction sites [16]. It is expected that there are favorable electrostatic interactions between CYPs and their electron transfer partner [17]. A crystal structure for a CYP-reductase-complex is not yet available. Even though the kinetics in P450 reduction may not be generalized among different P450 systems, and the concepts regarding the influence of a rate-limiting step are not universal [18], the electron transfer from the reductase to the heme domain is often slow and one of the rate-limiting aspects in many CYP systems [19]. However, the interactions between the components of the electron transfer systems still remain unclear. A deeper understanding of the factors determining reductase interaction gained by the analysis of the reductase interaction sites of CYPs will assist in improving interactions and consequently lead to optimized enzymes for biocatalytic applications [20].

Previous analyses of the structure conservation in CYPs showed that all CYPs have a well-conserved heme-binding structural core formed out of αD, αE, αI, and αL and αJ and αK [21]. The β-bulge region which contains the thiolate heme ligand is referred to as Cys-pocket. Between αK and the Cys-pocket, a structurally conserved region is located, the so-called 'meander' loop. It is spanned by 7-10 amino acid residues and is supposed to play a role in heme binding and stabilization of the tertiary structure. The proposed reductase interaction face of CYPs mainly comprises the αJ/αJ' and the insertion following the meander loop [6]. Since the structures of all CYPs are highly similar, but differ in substrate specificity and their electron transfer partners, the different biochemical properties of CYPs are mediated by the diverse regions, which vary in both sequence and structure [8].

Six regions which are involved in recognition and binding of substrates and hence determine substrate specificity were described as SRSs (substrate recognition sites [22]). SRS1 lies in the highly variable loop region between αB and αC (BC-loop), SRS2 is located in the C-terminal end of αF, SRS3 and SRS4 are spanned by the N-terminal regions of αG and αI, β1-4 houses SRS5 and β4-1 SRS6. While the access of the substrate to the binding pocket is limited by flexible regions in the entrance channel, such as αF and αG which undergo strong conformational changes upon substrate binding [23,24], the regions flanking directly the binding pocket and thus limiting the access of the substrate to the heme, namely αI, the BC-loop region and SRS5, were observed to remain rigid during simulation [25,26]. In a systematic analysis of SRS5 in more than 6300 sequences, single substrate- and heme-interacting residues could be identified in this region [27]: Thus, a hotspot for regio- and stereoselectivity in one residue in SRS5 and one position in the BC-loop (F87), were previously reported as key residues in determining activity, regio- and stereoselectivity in CYP102A1 [28-30]. Combinations of variants of these two positions were applied to design a minimal mutant library with improved selectivity [31]. Due to the high variability of the BC-loop, the identification of position 87 in CYP102A1 in other CYPs, remains a challenge for sequences without structural information.

To serve as a tool for a comprehensive comparison of protein sequences and structures within the vast and diverse family of CYPs in order to transfer the newly gained insights among the CYP sequences, the Cytochrome P450 Engineering Database (*CYPED*) [32] has been designed. In its current version 2.02 it contains 8614 sequences [33]. The highly similar structures have been compared in detail to identify the common core and to assign the variable regions. For this purpose a structural alignment was used as a base to generate a reliable structure profile. With this profile all structurally conserved regions (SCR) could be predicted and annotated among all *CYPED *protein sequence entries, hence allowing a structural navigation in those sequences lacking structural information. Beyond this, the *CYPED *website provides an interface which allows the prediction of the SCRs for every user-specified CYP sequence.

## Data

### CYP Structures

A set of 31 PDB structures [34] was extracted from version 1.1 of the *CYPED *[32] as listed in table 1. The selection includes 16 bacterial structures of class I and 12 CYPs assigned to class II CYPs, comprising CYPs which interact with a CPR-type FMN/FAD reductase. The structures in this class are predominantly of mammalian origin. The only exception is CYP102A1 (P450 BM-3) from *Bacillus megaterium*, which is a fusion enzyme, consisting of a P450 domain and a FMN/FAD reductase domain [15]. Because of its structural similarity to CYP102A1, the bacterial CYP175A1 isolated from the thermophilic *Thermus thermophilus *was also assigned to class II [14]. Additionally analyzed crystal structures were: CYP8A (human prostacyclin synthase), which accepts endoperoxides or hydroperoxides as substrates and does not require any electron-transfer partner or molecular oxygen [35]; CYP55A2 from *Fusarium oxisporum *and 152A1 from *Bacillus subtilis *(P450_Bsβ_) are representatives for CYPs which obtain electrons directly from NAD(P)H or catalyze a peroxide-dependent reaction. All structures represent the closed form of CYPs since including the open form as available for example for CYP2B4 [36] would worsen the alignment quality. Eleven recently published CYP structures were not included in the alignment but were used to validate the prediction of the structurally conserved regions.

**Table 1:** List of CYP structures analyzed in this work.

*CYP*	*PDB entry*	*Resolution [Å]*	*Ligand*	*Organism*
**Class II CYPs (CPR-type)**				

1A2	2HI4	1.95	+	*Homo sapiens*
2A6	1Z10	1.90	+	*Homo sapiens*
2A13	2P85	2.35	+	*Homo sapiens*
2B4	1SUO	1.90	+	*Oryctolagus cuniculus*
2C5	1N6B	2.30	+	*Oryctolagus cuniculus*
2C8	1PQ2	2.70	+	*Homo sapiens*
2C9	1OG2	2.60	+	*Homo sapiens*
2D6	2F9Q	3.00	-	*Homo sapiens*
2R1	2OJD	2.80	+	*Homo sapiens*
3A4	1TQN	2.05	-	*Homo sapiens*
102A1	1BU7	1.65	-	*Bacillus megaterium *(P450 BM-3)
175A1	1N97	1.80	-	*Thermus thermophilus*

**Class I CYPs**				

51B1	1E9X	2.10	+	*Mycobacterium tuberculosis*
101D	2CPP	1.63	+	*Pseudomonas putida *(P450cam)
107A1	1OXA	2.10	+	*Saccharopolyspora erythrea*
107L1	2BVJ	2.10	-	*Streptomyces venezuelae*
108A	1CPT	2.30	-	*Pseudomonas sp*.
119	1IO7	1.50	-	*Sulfolobus solfactaricus*
154A1	1ODO	1.85	+	*Streptomyces coelicolor*
154C1	1GWI	1.92	-	*Streptomyces coelicolor*
158A1	2DKK	1.97	+	*Streptomyces coelicolor*
158A2	1S1F	1.50	+	*Streptomyces coelicolor*
165B3	1LFK	1.70	-	*Amicolatopsis orientalis*
165C4	1UED	1.90	-	*Amicolatopsis orientalis*
167A1	1Q5D	1.93	+	*Polyangium cellulosum*
176A1	1T2B	1.70	+	*Citrobacter brakii*
199A2	2FR7	2.01	-	*Rhodopseudomonas palustris*
245A1	2Z3T	1.90	+	*Streptomyces sp*. TP-A0274

**Other CYPs**				

8A	2IAG	2.15	-	*Homo sapiens*
55A2	1CL6	1.70	+	*Fusarium oxisporum *(NO reductase)
152A1	1IZO	2.10	+	*Bacillus subtilis *(P450_Bsβ_)

Ligand-free and ligand-bound structures are indicated by - and +, respectively

### CYP Sequences

The analysis of CYP sequences and structures was performed based on the updated version 2.02 of the *CYPED *[33]. It integrates sequences of 8614 proteins. The proteins are organized into 249 superfamilies and 619 homologous families according to Nelson [4]. Reliable multisequence alignments are available for each family. The sequences are annotated by automatically extracted GenBank annotations [37], which were manually enriched. Secondary structure information is available as DSSP annotation within the multisequence alignments for those homologous families containing members with existing PDB structures.

## Methods

### Structure-based HMM profile

**Figure 1:**
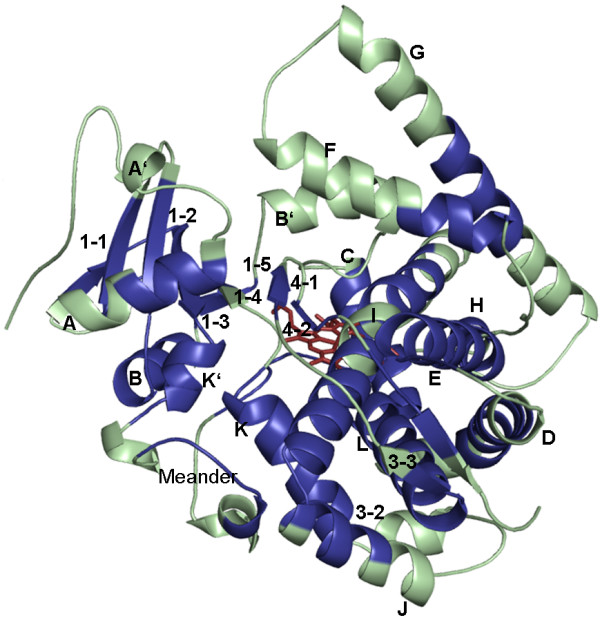
**SCRs of CYPs in a structural overview**. The structurally conserved regions were derived from the STAMP alignment and mapped on the reference structure of P450 BM-3 from *Bacillus megaterium *[PDB: 1BU7]. The SCRs are highlighted in blue, whereas the variable regions are shown in green.

SCRs were determined by the generation of a structure-based multisequence alignment using STAMP [38]. STAMP estimates the probability of structural equivalence of residues [39] and uses the Smith-Waterman algorithm [40] to determine the best path through a matrix of numerical pairwise similarity values of corresponding sequence positions. This allows STAMP to calculate two measures of alignment confidence: P'_ij_, a measure for residue equivalence and S_c_, the STAMP score, which reflects overall alignment quality. A S_c _> 5.5 implies a high degree of similarity of the considered structures. Stretches of residues in the alignment having P'_ij _> 6.0 imply regions of conserved secondary structure and are marked by black boxes in the alignment output. To visualize secondary structure information on the alignment output, STAMP uses DSSP [41] outputs. Therefore, in a first step DSSP was applied on the CYP structures to calculate secondary structure information. The resulting structure-based multisequence alignment was checked for correctly aligned secondary structures, ExxR motif and Cys-pocket. Regions with high P'_ij _which indicate conserved secondary structures were defined as SCR, extracted from the alignment and visualized (Figure 1) on the structure from CYP102A1 [PDB: 1BU7] as reference structure using PyMOL [42]. Structure-based HMM-profiles were derived from the structure-based multisequence alignments using HMMER http://hmmer.janelia.org/.

### Structural analysis

Structural superpositions and visualizations were generated using PyMOL [42]. The analysis of the BC-loop region was performed by an overall superposition of all structures on CYP102A1 [PDB: 1BU7]. The visualization of the reductase interaction sites RIS1 and RIS2 was generated by the superposition of the FMN-domains of CYP102A1 [PDB: 1BVY] and the CPR-type FMN/FAD reductase from *Rattus norvegicus *[PDB: 3ES9] and the superposition of the P450-domains of CYP2C9 from *Homo sapiens *[PDB: 1OG2] CYP101D and from *Pseudomonas putida *[PDB: 2CPP] on the P450-domain of CYP102A1.

### Sequence analysis

For the analysis of all CYP sequences, the *CYPED *and the *DWARF *system [43] were applied. The data warehouse system *DWARF *is the in-house repository for the *CYPED *data and assists local analysis. Besides integrating sequences and structures of this protein family, it provides a set of bioinformatics tools for sequence and structure analysis. We took advantage of its modular and extensible architecture and designed a Perl program which implements an automated procedure that subsequently generates a structure-based multisequence alignment for every *CYPED *entry by mapping it on the structure-based HMM profile which was derived from the STAMP alignment. Using the alignment row which represents the structure of CYP102A1 as a reference, the start and stop positions of each conserved secondary structure were identified within each alignment and transferred to the query sequence. Therefore, the absolute positions of the SCRs of each query sequence could be predicted. The positions were stored as annotations in the *CYPED *and are visualized in the multisequence alignments and on the feature page for each *CYPED *entry.

The same procedure as for the identification of the SCRs was applied to identify the specificity and regioselectivity determining position which corresponds to F87 in CYP102A1 in all sequences among the *CYPED*. Again, the sequence of the structure of CYP102A1 was used as the reference. Each *CYPED *query sequence was mapped on the structure-based HMM profile and the resulting alignment was used to determine the residue corresponding to F87.

The accuracy of this method was tested in a leave-one-out cross-validation [44] by generating for each of the 30 crystal structures a structure-based HMM profiles, leaving subsequently one structure out and mapping the sequence of the left-out crystal structure on the corresponding profile. The generated alignment was checked for the correct prediction of the residue corresponding to F87.

An online version of the prediction tool was integrated into the *CYPED *homepage. Since the method operates exclusively for sequences with CYP fold, input sequences are first checked for applicability by sequence homology via a BLAST [45] query using an E-value of 10^-100^. Structurally conserved regions are determined as described above.

## Results

### Structural Core

From the simultaneous superposition of the 31 structures using STAMP, a multiple sequence alignment could be derived which resulted in 257 structurally equivalent residues out of 400-450 residues. The calculated average RMS deviation after fitting all structures by these 257 residues was 2.4Å and their averaged sequence identity was 25%. The overall STAMP alignment score S_c _was 6.0 and is above the threshold for highly similar structures. Stretches of structurally equivalent residues (P'_ij _> 6.0) are marked by black boxes in the structure-based sequence alignment (figure S1, Additional file 1). The residues of the conserved core are organized into 19 SCRs that include at least partially all defined secondary structures αA-L and β1-4. The SCRs extracted from the structural alignment were mapped on the reference structure CYP102A1 from *Bacillus megaterium *(Figure 1).

**Figure 2:**
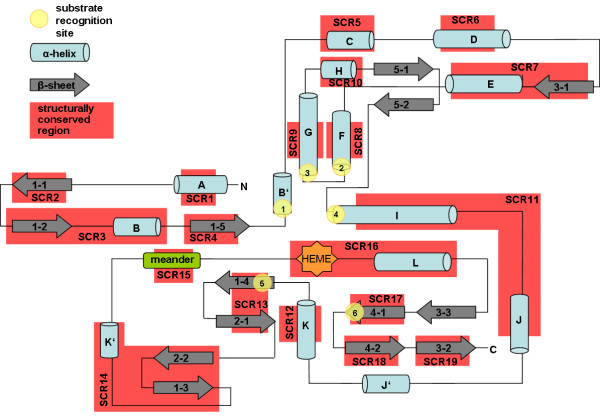
**SCRs of CYPs in a topological illustration**. The structurally conserved regions were derived from the STAMP alignment and their locations are marked in red. All α-helices are visualized as blue tubes, β-sheets as grey arrows and the SRS regions of CYPs are marked as yellow circles.

A topological overview of the conserved CYP structure illustrates the distribution of SCRs on the CYP structure (Figure 2). Some SCRs are part of individual secondary structures; other SCRs include several secondary structure elements. Among these, SCR3 comprises β1-2 and αB, SCR7 β3-1 and αE. SCR11 is assembled by αI and αJ and SCR13 by β1-4 and β2-1. β2-2, β1-3 and αK' together form SCR14 and the heme-binding Cys-pocket and αL together form SCR16. The structural alignment further revealed that the β-5 sheet which is not present in all CYP structures does not belong to the conserved parts of the CYP structures [14]. The variable termini of the secondary structure elements αF, αG, αI, β1-4, β4-1, and the BC-loop are surrounding the heme and house the residues defining the SRS regions 1-6.

By applying the procedure on each *CYPED *sequence and mapping it on the HMM profile generated from the STAMP alignment, the SCRs could be identified and annotated in all sequence entries. The conserved secondary structures appear in the online version of the *CYPED *either within the annotated multisequence alignments or on the feature page of each protein entry. Its labelling appears in moving over the respective region. The results of the online prediction for any CYP sequence are displayed as colored and annotated regions and as a tabular output listing each conserved secondary structure and the corresponding start and stop position.

### BC-loop

**Figure 3:**
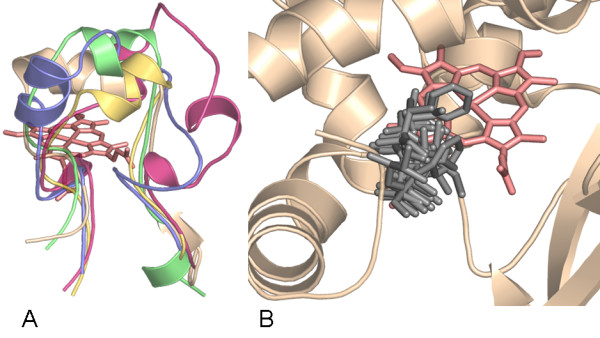
**BC-loop region (SRS1) of CYPs**. **(A) **Comparison of the BC-loops of P450 BM-3 [PDB: 1BU7] in beige, CYP2C9 [PDB: 1OG2] in green, CYP154C1 [PDB: 1GWI] in pink, CYP101D [PDB: 2CPP] in yellow and CYP107A1 [PDB: 1OXA] in blue. **(B) **BC-loop region in P450 BM-3 [PDB: 1BU7] and the position 87 corresponding residue in all 31 structures.

In CYP102A1, the phenylalanine at position 87 is assumed to mediate selectivity and activity. Due to its proximity to the heme center, this residue has a strong evidence to be involved in substrate binding and to control substrate specificity and regioselectivity [31]. Therefore, the identification of residues corresponding to this position would be beneficial in the design of CYPs with engineered properties. Since it is located in the SRS1 region of the highly variable BC-loop the identification of this position in enzymes without structural information is not possible merely by sequence alignment. However, a comprehensive analysis of the BC-loops in the structures analyzed in this work revealed that although being highly variable (Figure 3A), the BC-loop in almost every structure of different proteins that were compared houses one residue, which points directly towards the heme, and remains rigid during substrate binding, which could be shown by comparing multiple structures of the same protein (figures S2 and S3, Additional file 1). By the overall superposition of structures of different proteins on the structure of CYP102A1, it could be shown that this position is located exactly at the same position, corresponding to the phenylalanine in CYP102A1 (Figure 3B) located at position 87. Table 2 lists the corresponding residue in each structure.

**Table 2:** Positions which correspond to F87 in P450 BM-3 and predicted position, and prediction of positions in new structures

*CYP*	*PDB-Code*	*Position in crystal structure*	*Predicted position*
8A	2IAG	-	-
51B1	1E9X	-	V88
55A2	1CL6	V87	V87
101D	2CPP	T101	I99
107A1	1OXA	G91	G91
107L1	2BVJ	L93	L93
108A	1CPT	T103	T103
119	1IO7	L69	L69
152A1	1IZO	Q85	Q85
154A1	1ODO	F88	F88
154C1	1GWI	L93	L93
158A1	2DKK	A97	S95
158A2	1S1F	G94	G94
165B3	1LFK	M89	N87
165C4	1UED	S98	S98
167A1	1Q5D	F96	G94
175A1	1N97	L80	L80
176A1	1T2B	A91	M89
199A2	2FR7	L100	L100
245A1	2Z3T	V99	V99
1A2	2HI4	T124	S126
2A6	1Z10	V117	V117
2A13	2P85	A117	A117
2B4	1SUO	I114	I114
2C5	1N6B	A113	A113
2C8	1PQ2	I113	I113
2C9	1OG2	V113	V113
2D6	2F9Q	F120	F120
2R1	2OJD	L125	L125
3A4	1TQN	S119	S119

**102A1**	1BU7**(reference)**	**F87**	**F87**

2E1	3E4E	I94	I94
3A43	2V0M	S119	S119
7A1	2DAX	-	D98
19A1	3EQM	F134	F134
46A1	2Q9F	V126	S127
74A1	2RCH	S128	L127
105A1	2ZBX	I96	I96
105K1	2Z36	L96	L96
120A1	2VE3	A94	A94
231A2	2RFB	I48	I48
248A	3BUJ	L80	L80

To validate our structure-based method to assign SCRs in a one-leave-out cross-validation, the position which corresponds to F87 in CYP102A1 was predicted for each sequence of each structure. For 23 out of 30 (80%), the predicted positions agreed with the crystal structure, in 7 CYPs they deviated by up to 2 residues. To further apply and to validate the procedure, the position was predicted in eleven structures published in progress of this study. 8 correct predictions, 2 deviations by one position, and one wrong prediction for the case of CYP7A1 which has in the crystal structure no residue located at this position, again confirmed an accuracy of 80%. It should be noticed that in some crystal structures the residue numbering of the structures deviates from the residue numbering in the sequences due to missing residues and therefore the numbering of the protein structure was considered. The crystal structure of CYP231A from the thermoacidophilic *Picrophilus torridus *was missing a part of the BC-loop [46] which made the prediction not clearly defined.

### Amino acid composition of the F87 corresponding position

**Figure 4:**
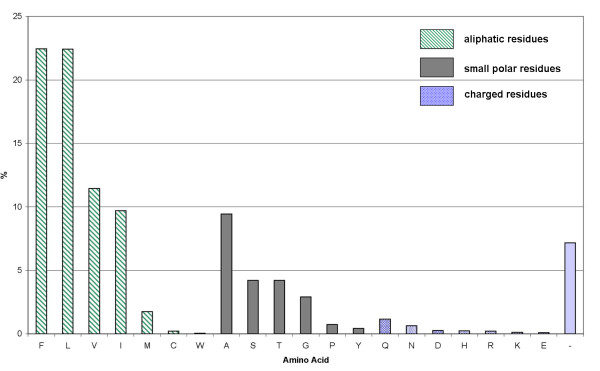
**Residues at F87 corresponding position**. Amino acid composition of predicted F87 corresponding positions in all 8614 *CYPED *proteins. Green bars correspond to the percentage of aliphatic residues and phenylalanine, grey bars to amino acids of small polar nature and blue bars to charged amino acids. '-' denotes a gap.

In addition to the identification of the F87 corresponding position, a comprehensive analysis of the sequences of all 8614 CYPED protein entries was performed in respect to the amino acid composition, by a prediction of the position in all sequences analogous to the SCR prediction. It could be observed that 73% of the residues predicted at this position include aliphatic residues and phenylalanine. The remaining 24% at this position are small polar residues and only are 3% charged residues. Phenylalanine (22%), leucine (22%), and valine (12%) were the most frequently occuring amino acids followed by isoleucine (10%) and alanine (9%). Other amino acids appear more rarely with frequencies less than 4% (Figure 4). A predicted gap at this position indicates that the BC-loop region houses no residue which is located close to the heme or the BC-loop itself winds away from the active site as it could be observed for example in the structures CYP8A and CYP51B1.

### Analysis of reductase interaction sites

**Figure 5:**
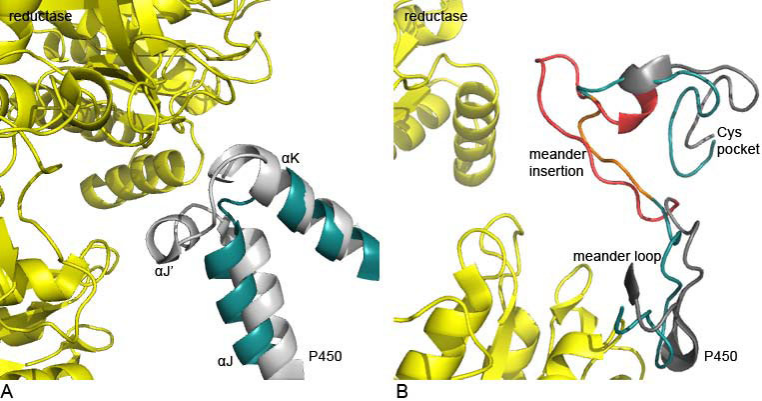
**Sites interacting with potential redox partners**. The CPR-type FMN/FAD from *Rattus norvegicus *[PDB: 3ES9] is shown in yellow, the parts of the P450 domain of CYP2C9 from *Homo sapiens *[PDB: 1OG2] are shown in grey and green for CYP101D from *Pseudomonas putida *[PDB: 2CPP], respectively. **(A) **Comparison RIS1 (αJ/J' region) of the human CYP2C9 and P450cam CYP101D. **(B) **Comparison of RIS2 (meander insertion) of the human CYP2C9 and P450cam CYP101D.

The structural regions αJ/J' and the insertion between the meander loop and the Cys-pocket are of particular interest since they were previously proposed to form the reductase interacting face of the molecules [6]. These sites strongly vary in their length and conformation. The structural analysis (Figure 5) reflects the differences of αJ/J' (further referred to as reductase interaction site 1, RIS1) (Figure 5A) and the insertion between meander loop and Cys-pocket (further referred to as reductase interaction site 2, RIS2) (Figure 5B) of CYPs from different redox classes. A comparison of the human CYP2C9 and the bacterial P450cam CYP101D shows that RIS1 (αJ/J' region) of CYP2C9 is 18 residues longer. RIS2 differs by 9 residues between CYP2C9 and CYP101D. By counting the number of residues spanning these regions in the STAMP alignment (figure S1, Additional file 1), it was revealed that these regions in class II CYPs interacting with CPR-type reductases are long, in class I CYPs extremely short or not existing at all and that those CYPs which do not require any electron transfer partner form a subgroup of class II, in some cases with extremely long loops. The αJ/J' region differs from 21 to 22 residues for class II (long) and 3 to 5 residues for class I CYPs (short). The length of the meander insertion differs from 11 to 17 residues for class II (long), up to 23 residues (very long) in those CYPs which do not require a redox partner and 3 to 5 residues for class I CYPs (short).

**Figure 6:**
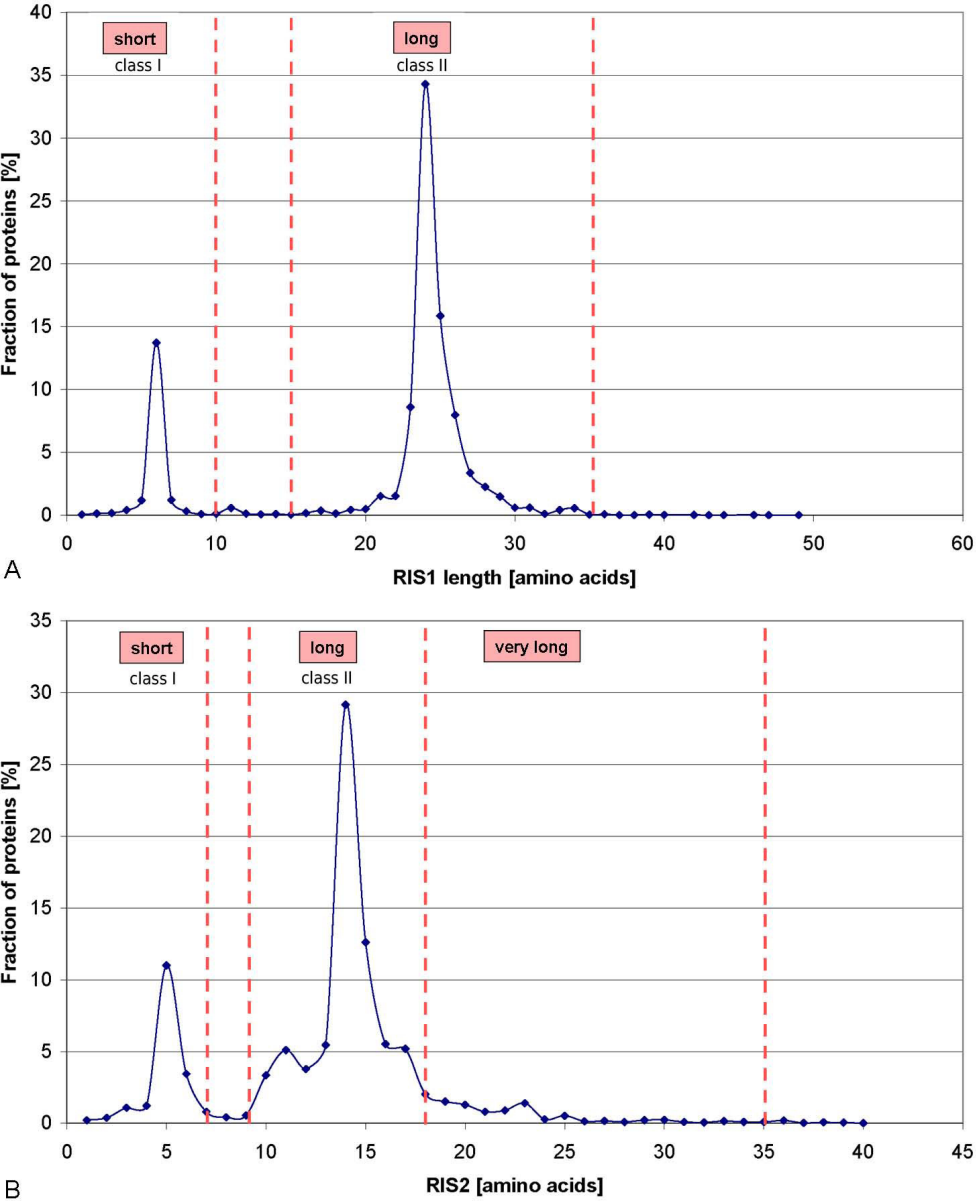
**Analysis of RIS length**. **(A) **Fraction of proteins for each RIS1 (αJ/J' region) length. **(B) **Fraction of proteins for each RIS2 (meander insertion) length.

Counting the number of amino acids in each *CYPED *sequence for RIS1 (Figure 6A) revealed two peaks in the RIS1 length distribution. This allowed defining two classes. Proteins having short RIS1 with less than 10 residues spanning the αJ/J' region make up 17.5% of all protein entries. According to the result of the length analysis of RIS1 of the structural alignment, they comprise class I CYPs. Proteins having long RIS1 with more than 15 residues spanning the αJ/J' region make up 81% of all protein entries. According to the result of the length analysis of RIS1 of the structural alignment, they comprise class II CYPs. Only 1% of all protein entries can not reliably be assigned by RIS1 length since their length is in between 10 and 15 amino acids.

The analysis of the length of RIS2 in each *CYPED *sequence (Figure 6B) showed a distribution in three main areas. Therefore, three classes according to the result of the length analysis of RIS2 in the structural alignment were defined. Proteins having short RIS2 with less than 7 residues spanning the meander insertion make up 18% of all protein entries. According to the result of the length analysis of RIS2 in the structural alignment, they comprise class I CYPs. Proteins having long RIS2 with between 11 and 17 residues spanning the meander insertion make up 66% of all protein entries. According to the result of the length analysis of RIS2 in the structural alignment, they comprise class II CYPs, with a subgroup of proteins having very long RIS2 with more than 18 residues spanning the meander insertion. 4% of all protein entries can not reliably be assigned by RIS2 length since their length is in between 8 and 10 amino acids.

0.5% of entries with RIS1 and 0.5% of entries with RIS2 length above 35 amino acids were formally assigned to class II, but could not be further analyzed since they comprise biochemically not characterized proteins.

## Discussion

Despite their inherently low sequence similarity, all CYPs share a common structural fold. The well-defined secondary structure elements can be found in all determined crystal structures, which house their active-site with the cofactor heme deeply inside the protein [21]. The generation of a structural alignment out of 31 CYP structures revealed structurally conserved regions which contain most of the described secondary structure elements of the CYP fold. It could be shown that some of the secondary structure elements merge together to structure modules, described as structurally conserved regions (SCR) 1-19, reflecting the modular structure of cytochrome P450 monooxygenases. The generation of a reliable structure-based HMM profile which was applied to every *CYPED *entry assisted in consistently annotating the conserved secondary structures in the *CYPED *entries. But besides addressing the problem of predicting conserved regions, an even more challenging issue could be solved: the identification and classification of the variable regions.

Since the residues that determine the substrate specificity of CYPs are assumed to lie in the variable regions [8,22], their identification is of greatest interest for engineering of biochemical properties. Two of the six proposed substrate recognition sites, SRS1 and SRS5, together with the helix I directly flank the substrate binding cavity and are therefore supposed to interact with the substrate [27]. SRS1 houses a residue, which previously was described as essential for activity, regio- and stereoselectivity in CYP102A1 [28-30]. Located at position 87 and pointing directly towards the heme, a corresponding residue to this phenylalanine can be found in almost all CYP structures. Its location in the highly variable BC-loop region makes its determination very difficult in sequences without structural information.

The position, which corresponds to F87 in CYP102A1 could be correctly predicted in almost 80% of all analyzed CYP structures. By surveying more recent CYP structures, the validity of the prediction could be confirmed. The analysis of this position in all 8614 CYP sequences in the *CYPED *revealed that the residues at this position predominantly are of aliphatic nature or a phenylalanine, less frequently small polar amino acids and only very infrequently of charged nature. Since the characteristics of the residue at this position highly influence substrate specificity and regioselectivity, its identification contributes to the design of CYPs with more suitable properties for biocatalytic applications.

Even though there were two reductase interaction sites proposed to be located in αJ/αJ' and in the insertion following the meander loop [6], termed RIS1 and RIS2, these regions which are highly variable in sequence and structure were difficult to determine in sequences. The identification of the preceding and the successive SCR solved this problem. Depending on the length for RIS1, two classes (short and long RIS1) and three classes for RIS2 (short, long and very long RIS2) were introduced. From the analysis of the CYP structures in respect to their redox partner it was assumed that class II CYPs have long RIS1 and long RIS2, class I CYPs have short RIS1 and short RIS 2.

The largest percentage of all CYPs has long RIS1 and long RIS2 (53%). All CYPs with available structure which possess these long loops clearly belong to class II, and most of them are of human origin. The class II protein P450 BM-3 also shows the characteristic CPR-interacting loop length. The 12% of proteins with short RIS1 and RIS2, respectively, are assumed to be class I proteins. 27% could not be clearly classified, either because of unusual long loops (above 35 residues), or a combination of short RIS1 with long RIS2 and vice versa. This comparison of reductase interaction sites allows drawing conclusions on its reductase interaction.

The remaining 8% of CYPs consist of proteins with long RIS1 and very long RIS2. Members of this unusual group cannot easily be categorized in regards to their reductase interaction. For example, the human prostacyclin synthase CYP8A1, which has endoperoxidase activity and does not require a reductase as source of electrons, is a representative of this class of proteins [35]. It has a long RIS1, consisting of 22 amino acids and a very long RIS2 of 23 amino acids. However, the crystal structure for the human cholesterol 7 alpha-hydroxylase CYP7A1 which was recently solved also contains very long proximal loops [47] that were correctly predicted containing 22 (RIS1) and 23 (RIS2) amino acids. CYP7A1 was previously compared to the structure of CYP8A1 [48], but in contrast is a typical monooxygenase.

The fatty acid hydroxylase CYP152A1 from *Bacillus subtilis *(P450_Bsβ_) is a hydrogen peroxide driven enzyme [49] and therefore could be assigned to those CYPs which do not require a redox partner. CYP152A1 has a short RIS1 of 5 amino acid residues and a long RIS2 of 11 residues, like the CPR-type interacting class II CYPs, which is unexpected for this kind of CYP. Indeed, CYP152A1 and its homologous protein CYP152A2 from *Clostridium acetobutylicum *(P450_CLA_) experimentally showed much higher conversions in the presence of a CPR-type reductase than in the presence of hydrogen peroxide and the absence of a reductase [50]. The recently solved crystal structure of the allene oxide synthase CYP74A1 is an atypical cytochrome P450 family member and does not require a reductase [51]. However, CYP74A1 also shows similar loop lengths to class II CYPs of RIS1 of 21 AS and RIS2 of 10 AS. Due to an unusual nine amino acid insert in the Cys-pocket which allows its access to the protein surface, the interaction with a redox partner might be disrupted [51]. Therefore, CYP74A1 cannot be compared to typical monooxygenases with similar RIS1 and RIS2 length by our model.

Since most CYPs require electrons from a redox partner, and CYP152A1 and CYP152A2 showed higher activities by adding a reductase, it can be assumed that the interaction of CYPs with reductases plays a pivotal role in the CYP mechanism. Finding the optimal redox partner for CYPs may significantly enhance their activity but is quite difficult. The analysis and classification which led to the prediction of possible redox partner interactions offers the potential of engineering enhanced interactions.

## Conclusion

In order to navigate in CYP sequences and to determine functionally relevant residues, a procedure which allows identifying conserved modules and functionally relevant sites within variable regions was implemented. Regions involved in substrate binding as well as redox partner recognition and interaction could be determined in the absence of structural information, based on sequence only. The structurally annotated sequences and multisequence alignments are accessible on the current version of the *CYPED *http://www.cyped.uni-stuttgart.de. Via a web interface integrated in the *CYPED *homepage at http://www.cyped.uni-stuttgart.de/cgi-bin/strpred/dosecpred.pl, the structural prediction is provided for every sequence which is similar to CYPs or presumably shares the CYP fold. The navigation in CYP sequences and the determination of functionally relevant sites in turn is a great advantage in the prediction of promising targets for the design of CYPs with improved biocatalytic properties.

## Authors' contributions

DS implemented the program, performed the analysis and wrote the manuscript. MW contributed to the analysis and to the manuscript. FW carried out the annotation and generated the web interface. JP supervised the project and finalized the manuscript. All authors read and approved the final version of the manuscript.

## List of abbreviations

CYP: Cytochrome P450 monooxygenase; CYPED: Cytochrome P450 Engineering Database; CPR: Cytochrome P450 reductase; P450 BM-3: Cytochrome P450 monooxygenase BM-3 from *Bacillus megaterium*; DWARF: Data Warehouse for Analyzing Protein Families; BLAST: Basic Local Alignment Search Tool; HMM:Hidden Markov model; STAMP: Structural Alignment of Multiple Proteins; DSSP: Define Secondary Structure of Proteins; SRS: Substrate recognition site; SCR: Structurally conserved region; RIS: Reductase interaction site.

## Supplementary Material

Additional file 1 **This file contains figures S1, S2, and S3 mentioned in the text**. Click here for file

## References

[B1] Ortiz de Montellano PR Cytochrome P450: structure, mechanism and biochemistry 1995 New York, Plenum Press

[B2] Raucy JL Allen SW Recent advances in P450 research Pharmacogenomics J 2001 1 178 186 11908754 10.1038/sj.tpj.6500044

[B3] Urlacher VB Eiben S Cytochrome P450 monooxygenases: perspectives for synthetic application Trends Biotechnol 2006 24 324 330 10.1016/j.tibtech.2006.05.002 16759725

[B4] Nelson DR Cytochrome P450 nomenclature, 2004 Methods Mol Biol 2006 320 1 10 16719369 10.1385/1-59259-998-2:1

[B5] Graham SE Peterson JA How similar are P450s and what can their differences teach us? Arch Biochem Biophys 1999 369 24 29 10.1006/abbi.1999.1350 10462437

[B6] Hasemann CA Kurumbail RG Boddupalli SS Peterson JA Deisenhofer J Structure and function of cytochromes P450: a comparative analysis of three crystal structures Structure 1995 3 41 62 10.1016/S0969-2126(01)00134-4 7743131

[B7] de Graaf C Vermeulen NPE Feenstra KA Cytochrome p450 in silico: an integrative modeling approach J Med Chem 2005 48 2725 2755 10.1021/jm040180d 15828810

[B8] Peterson JA Graham SE A close family resemblance: the importance of structure in understanding cytochromes P450 Structure 1998 6 1079 1085 10.1016/S0969-2126(98)00109-9 9753700

[B9] Werck-Reichhart D Feyereisen R Cytochromes P450: a success story Genome Biol 2000 1 REVIEWS3003 10.1186/gb-2000-1-6-reviews3003 11178272 PMC138896

[B10] Hannemann F Bichet A Ewen KM Bernhardt R Cytochrome P450 systems-biological variations of electron transport chains Biochim Biophys Acta 2007 1770 330 344 16978787 10.1016/j.bbagen.2006.07.017

[B11] McLean KJ Sabri M Marshall KR Lawson RJ Lewis DG Clift D Balding PR Dunford AJ Warman AJ McVey JP Biodiversity of cytochrome P450 redox systems Biochem Soc Trans 2005 33 796 801 10.1042/BST0330796 16042601

[B12] Munro AW Girvan HM McLean KJ Cytochrome P450-redox partner fusion enzymes Biochim Biophys Acta 2007 1770 345 359 17023115 10.1016/j.bbagen.2006.08.018

[B13] Roberts GA Grogan G Greter A Flitsch SL Turner NJ Identification of a new class of cytochrome P450 from a Rhodococcus sp Journal of Bacteriology 2002 184 3898 3908 10.1128/JB.184.14.3898-3908.2002 12081961 PMC135161

[B14] Baudry J Rupasinghe S Schuler MA Class-dependent sequence alignment strategy improves the structural and functional modeling of P450s Protein Eng Des Sel 2006 19 345 353 10.1093/protein/gzl012 16777908

[B15] Munro AW Leys DG McLean KJ Marshall KR Ost TWB Daff S Miles CS Chapman SK Lysek DA Moser CC P450 BM3: the very model of a modern flavocytochrome Trends Biochem Sci 2002 27 250 257 10.1016/S0968-0004(02)02086-8 12076537

[B16] Bernhardt R Cytochrome P450: structure, function, and generation of reactive oxygen species Rev Physiol Biochem Pharmacol 1996 127 137 221 full_text 8533008 10.1007/BFb0048267

[B17] Wade RC Motiejunas D Schleinkofer K Sudarko Winn PJ Banerjee A Kariakin A Jung C Multiple molecular recognition mechanisms. Cytochrome P450-a case study Biochim Biophys Acta 2005 1754 239 244 16226496 10.1016/j.bbapap.2005.07.044

[B18] Guengerich FP Johnson WW Kinetics of ferric cytochrome P450 reduction by NADPH-cytochrome P450 reductase: Rapid reduction in the absence of substrate and variations among cytochrome P450 systems Biochemistry 1997 36 14741 14750 10.1021/bi9719399 9398194

[B19] Guengerich FP Rate-limiting steps in cytochrome P450 catalysis Biol Chem 2002 383 1553 1564 10.1515/BC.2002.175 12452431

[B20] Bernhardt R Cytochromes P450 as versatile biocatalysts J Biotechnol 2006 124 128 145 10.1016/j.jbiotec.2006.01.026 16516322

[B21] Mestres J Structure conservation in cytochromes P450 Proteins 2005 58 596 609 10.1002/prot.20354 15617063

[B22] Gotoh O Substrate recognition sites in cytochrome P450 family 2 (CYP2) proteins inferred from comparative analyses of amino acid and coding nucleotide sequences J Biol Chem 1992 267 83 90 1730627

[B23] Li HY Poulos TL The structure of the cytochrome p450BM-3 haem domain complexed with the fatty acid substrate, palmitoleic acid Nature Structural Biology 1997 4 140 146 10.1038/nsb0297-140 9033595

[B24] Li HY Poulos TL Fatty acid metabolism, conformational change, and electron transfer in cytochrome P-450(BM-3) Biochimica Et Biophysica Acta-Molecular and Cell Biology of Lipids 1999 1441 141 149 10.1016/S1388-1981(99)00161-4 10570242

[B25] Branco RJF Seifert A Budde M Urlacher VB Ramos MJ Pleiss J Anchoring effects in a wide binding pocket: The molecular basis of regioselectivity in engineered cytochrome P450 monooxygenase from B. megaterium Proteins-Structure Function and Bioinformatics 2008 73 597 607 10.1002/prot.22083 18473391

[B26] Seifert A Tatzel S Schmid RD Pleiss J Multiple molecular dynamics simulations of human P450 monooxygenase CYP2C9: The molecular basis of substrate binding and regioselectivity toward warfarin Proteins-Structure Function and Bioinformatics 2006 64 147 155 10.1002/prot.20951 16639745

[B27] Seifert A Pleiss J Identification of selectivity-determining residues in cytochrome P450 monooxygenases: a systematic analysis of the substrate recognition site 5 Proteins 2009 74 1028 1035 10.1002/prot.22242 18814300

[B28] Li HM Mei LH Urlacher VB Schmid RD Cytochrome P450 BM-3 evolved by random and saturation mutagenesis as an effective indole-hydroxylating catalyst Appl Biochem Biotechnol 2008 144 27 36 10.1007/s12010-007-8002-5 18415984

[B29] Urlacher V Schmid RD Biotransformations using prokaryotic P450 monooxygenases Curr Opin Biotechnol 2002 13 557 564 10.1016/S0958-1669(02)00357-9 12482514

[B30] Urlacher VB Makhsumkhanov A Schmid RD Biotransformation of beta-ionone by engineered cytochrome P450 BM-3 Appl Microbiol Biotechnol 2006 70 53 59 10.1007/s00253-005-0028-4 16001257

[B31] Seifert A Vomund S Grohmann K Kriening S Urlacher VB Laschat S Pleiss J Rational design of a minimal and highly enriched CYP102A1 mutant library with improved regio-, stereo- and chemoselectivity Chembiochem 2009 10 853 861 10.1002/cbic.200800799 19222039

[B32] Fischer M Knoll M Sirim D Wagner F Funke S Pleiss J The Cytochrome P450 Engineering Database: a navigation and prediction tool for the cytochrome P450 protein family Bioinformatics 2007 23 2015 2017 10.1093/bioinformatics/btm268 17510166

[B33] Sirim D Wagner F Lisitsa A Pleiss J The cytochrome P450 engineering database: Integration of biochemical properties BMC Biochem 2009 10 27 10.1186/1471-2091-10-27 19909539 PMC2779185

[B34] Berman HM Battistuz T Bhat TN Bluhm WF Bourne PE Burkhardt K Feng Z Gilliland GL Iype L Jain S The Protein Data Bank Acta Crystallogr D Biol Crystallogr 2002 58 899 907 10.1107/S0907444902003451 12037327

[B35] Chiang C Yeh H Wang L Chan N Crystal Structure of the Human Prostacyclin Synthase J Mol Biol 2006 364 266 274 10.1016/j.jmb.2006.09.039 17020766 PMC1995163

[B36] Scott EE He YA Wester MR White MA Chin CC Halpert JR Johnson EF Stout CD An open conformation of mammalian cytochrome P4502B4 at 1.6-angstrom resolution Proceedings of the National Academy of Sciences of the United States of America 2003 100 13196 13201 10.1073/pnas.2133986100 14563924 PMC263748

[B37] Benson DA Karsch-Mizrachi I Lipman DJ Ostell J Wheeler DL GenBank Nucleic Acids Res 2008 36 D25 D30 10.1093/nar/gkm929 18073190 PMC2238942

[B38] Russell RB Barton GJ Multiple protein sequence alignment from tertiary structure comparison: assignment of global and residue confidence levels Proteins 1992 14 309 323 10.1002/prot.340140216 1409577

[B39] Rossmann MG Argos P Exploring structural homology of proteins J Mol Biol 1976 105 75 95 10.1016/0022-2836(76)90195-9 186608

[B40] Smith TF Waterman MS Identification of common molecular subsequences J Mol Biol 1981 147 195 197 10.1016/0022-2836(81)90087-5 7265238

[B41] Kabsch W Sander C Dictionary of protein secondary structure: pattern recognition of hydrogen-bonded and geometrical features Biopolymers 1983 22 2577 2637 10.1002/bip.360221211 6667333

[B42] Delano WL The PyMOL Molecular Graphics System San Carlos, CA, USA: DeLano Scientific 2002

[B43] Fischer M Thai QK Grieb M Pleiss J DWARF-a data warehouse system for analyzing protein families BMC Bioinformatics 2006 7 495 10.1186/1471-2105-7-495 17094801 PMC1647292

[B44] Picard RR Cook RD Cross-Validation of Regression Models Journal of the American Statistical Association 1984 79 575 583 10.2307/2288403

[B45] Altschul SF Madden TL Schaeffer AA Zhang J Zhang Z Miller W Lipman DJ Gapped BLAST and PSI-BLAST: a new generation of protein database search programs Nucleic Acids Res 1997 25 3389 3402 10.1093/nar/25.17.3389 9254694 PMC146917

[B46] Ho WW Li H Nishida CR de Montellano PRO Poulos TL Crystal structure and properties of CYP231A2 from the thermoacidophilic archaeon Picrophilus torridus Biochemistry 2008 47 2071 2079 10.1021/bi702240k 18197710

[B47] Strushkevich NV Tempel W Dombrovski L Dong A Loppnau P Arrowsmith CH Edwards AM Bountra C Wilkstrom M Bochkarev A Park H Crystal structure of human CYP7A1 To be Published

[B48] Mast N Graham SE Andersson U Bjorkhem I Hill C Peterson J Pikuleva IA Cholesterol binding to cytochrome P450 7A1, a key enzyme in bile acid biosynthesis Biochemistry 2005 44 3259 3271 10.1021/bi047566a 15736936

[B49] Lee DS Yamada A Sugimoto H Matsunaga I Ogura H Ichihara K Adachi SI Park SY Shiro Y Substrate recognition and molecular mechanism of fatty acid hydroxylation by cytochrome P450 from Bacillus subtilis. Crystallographic, spectroscopic, and mutational studies J Biol Chem 2003 278 9761 9767 10.1074/jbc.M211575200 12519760

[B50] Girhard M Schuster S Dietrich M Dürre P Urlacher VB Cytochrome P450 monooxygenase from Clostridium acetobutylicum: a new alpha-fatty acid hydroxylase Biochem Biophys Res Commun 2007 362 114 119 10.1016/j.bbrc.2007.07.155 17706598

[B51] Lee DS Nioche P Hamberg M Raman CS Structural insights into the evolutionary paths of oxylipin biosynthetic enzymes Nature 2008 455 363 U327 10.1038/nature07307 18716621

