# Correction: Prediction and analysis of the modular structure of cytochrome P450 monooxygenases

**DOI:** 10.1186/1472-6807-12-4

**Published:** 2012-04-25

**Authors:** Demet Sirim, Michael Widmann, Florian Wagner, Jürgen Pleiss

**Affiliations:** 1 Institute of Technical Biochemistry, University of Stuttgart, Allmandring 31, 70569, Stuttgart, Germany

## Correction

In our article (Sirim et al. BMC Struct Biol 2010, 10: 34) [1] the cytochrome P450 monooxygenase P450cam was referred to as CYP101D. In the latest release 3.0 of our cytochrome P450 database CYPED (http://www.CYPED.uni-stuttgart.de) P450cam was reassigned to family CYP101A and referred to as CYP101A1.
