# Supplementary material for: Prediction and analysis of the modular structure of cytochrome P450 monooxygenases
Source: BMC Struct Biol. 2010 Oct 15;10:34. doi: 10.1186/1472-6807-10-34 (PMC3224734; doi:10.1186/1472-6807-10-34)
Supplement: Additional file 1 — This file contains figures S1, S2, and S3 mentioned in the text. [file 1472-6807-10-34-S1.PDF]

Prediction and analysis of the modular structure of cytochrome P450 monooxygenases

Demet Sirim, Michael Widmann, Florian Wagner, Jürgen Pleiss[§]

Supplementary material

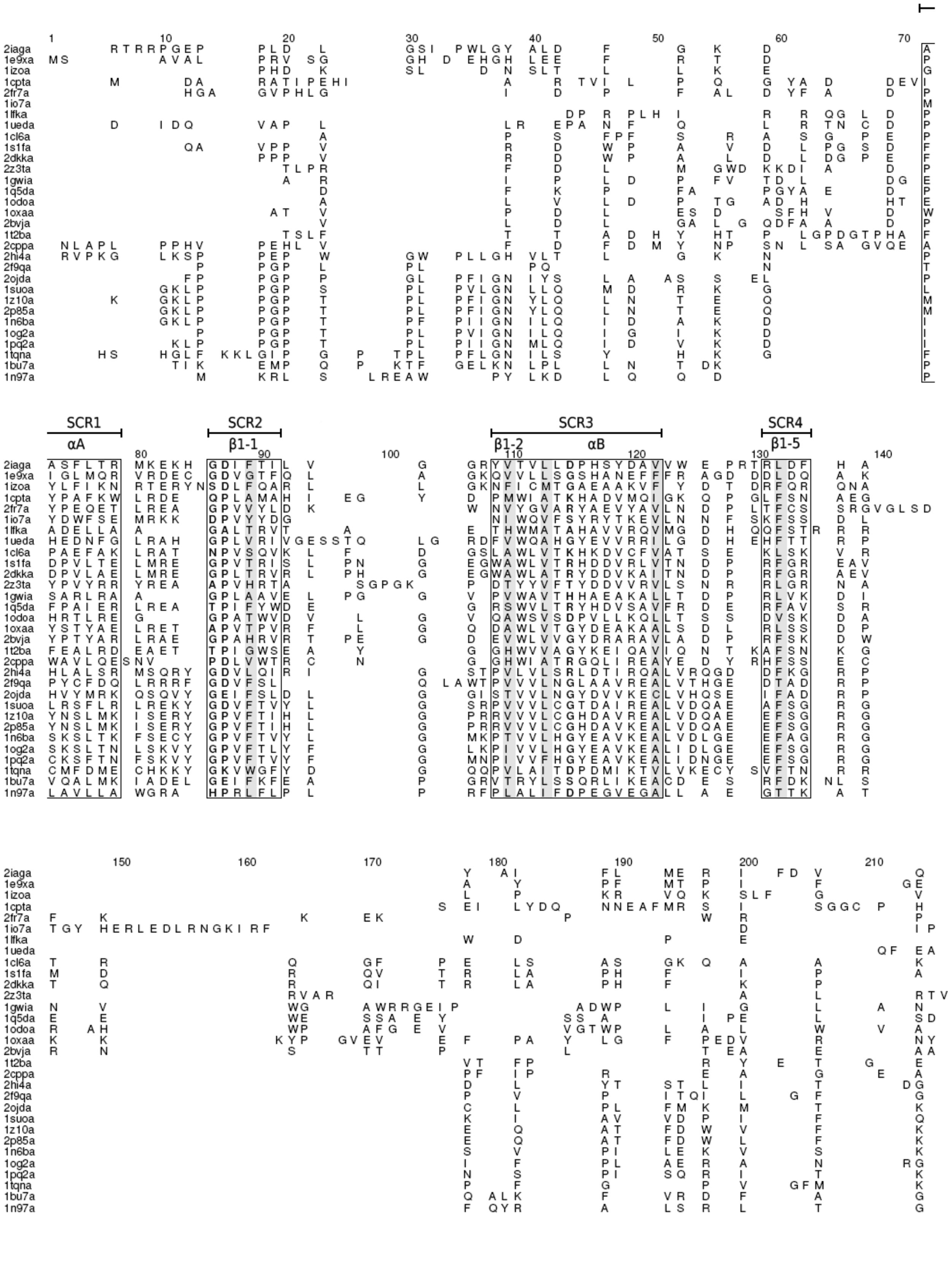

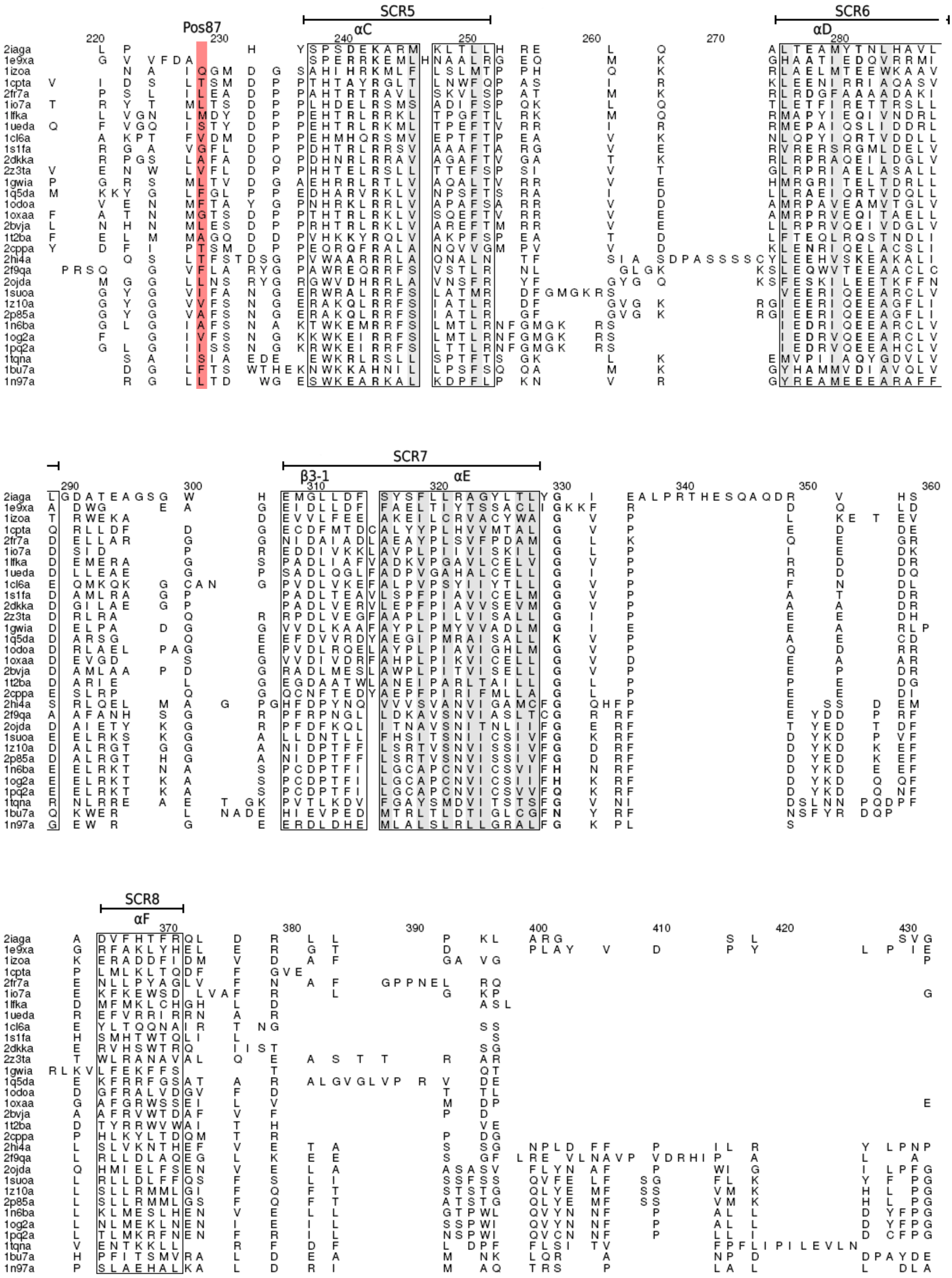

Pos87
SCR5
αC
SCR6
αD
SCR7
β3-1
αE
SCR8
αF
2iaga
1e9xa
1izoa
1cpta
2fr7a
1io7a
1lfka
1ueda
1cl6a
1s1fa
2dkka
2z3ta
1gwia
1q5da
1odoa
1oxaa
2bvja
1t2ba
2cppa
2hi4a
2f9qa
2ojda
1suoa
1z10a
2p85a
1n6ba
1og2a
1pq2a
1tqna
1bu7a
1n97a

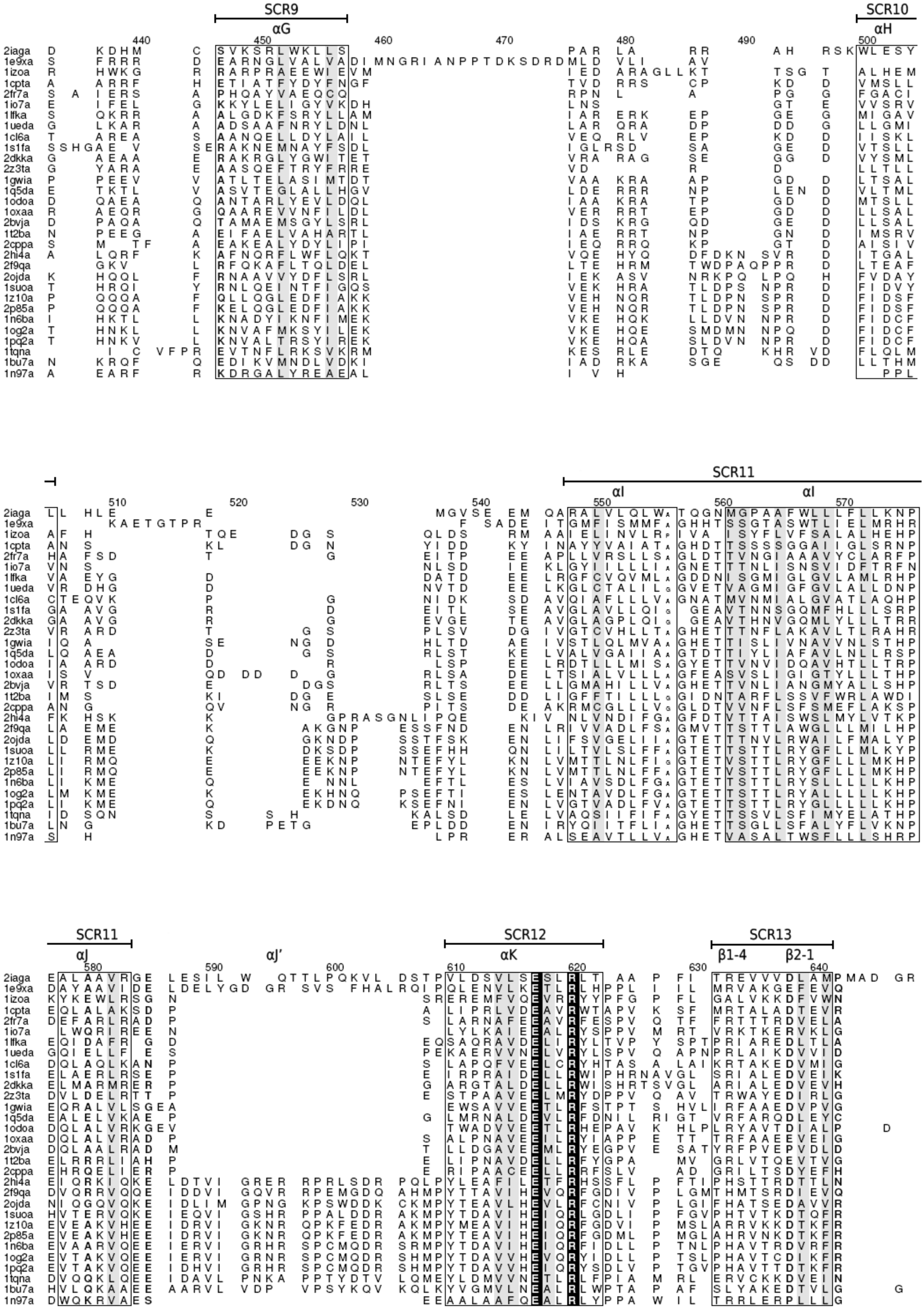
SCR9
αG
SCR10
αH
SCR11
αI
αI
SCR11
αJ
αJ'
SCR12
αK
SCR13
β1-4
β2-1

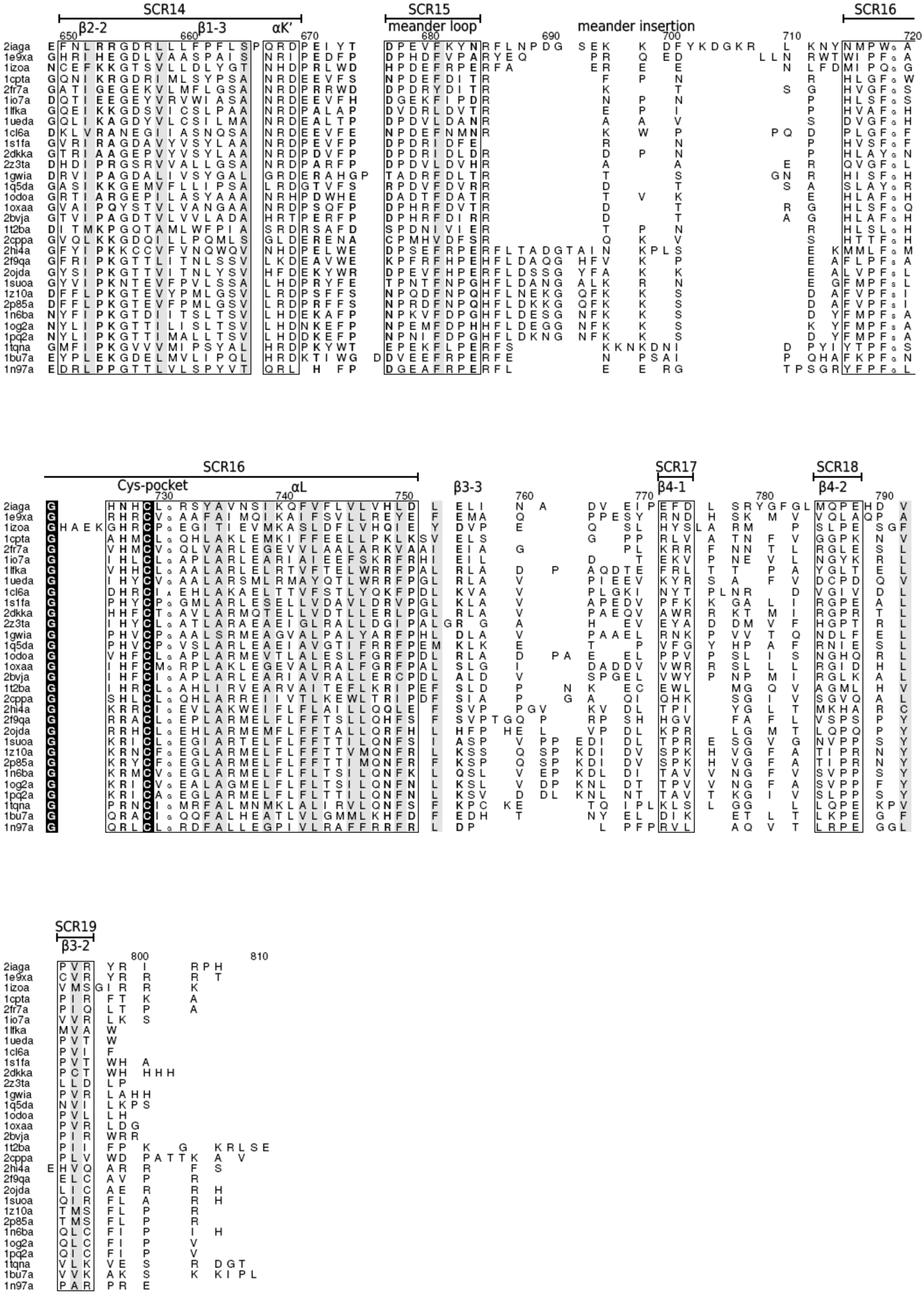

Figure S1 - Structure-based multisequence alignment of 31 CYP structures.

Regions marked by black boxes in the alignment have high P'_{ij} and imply regions of conserved secondary structure. Pos87 and the corresponding residues are highlighted in red.

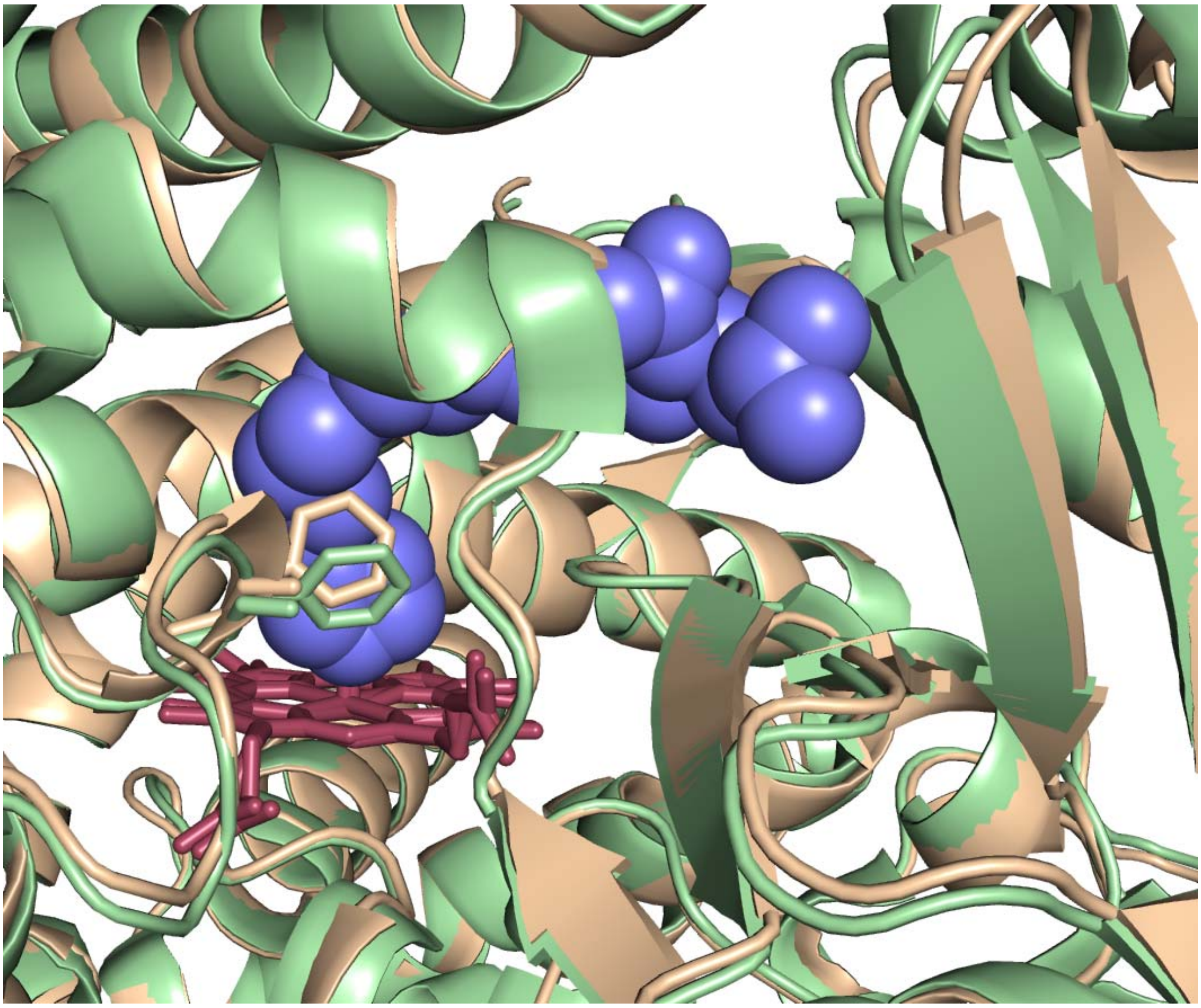

Figure S2 - Comparison of substrate-bound and substrate-free structure of CYP102A1 (P450 BM-3)

The substrate free structure is shown in wheat [PDB: 1BU7]. The substrate-bound structure [PDB: 3BEN] in green (with bound inhibitor N-[12-(1H-imidazol-1-yl)dodecanoyl]-L-leucine in blue). The phenylalanines at positions 87 are represented as sticks.

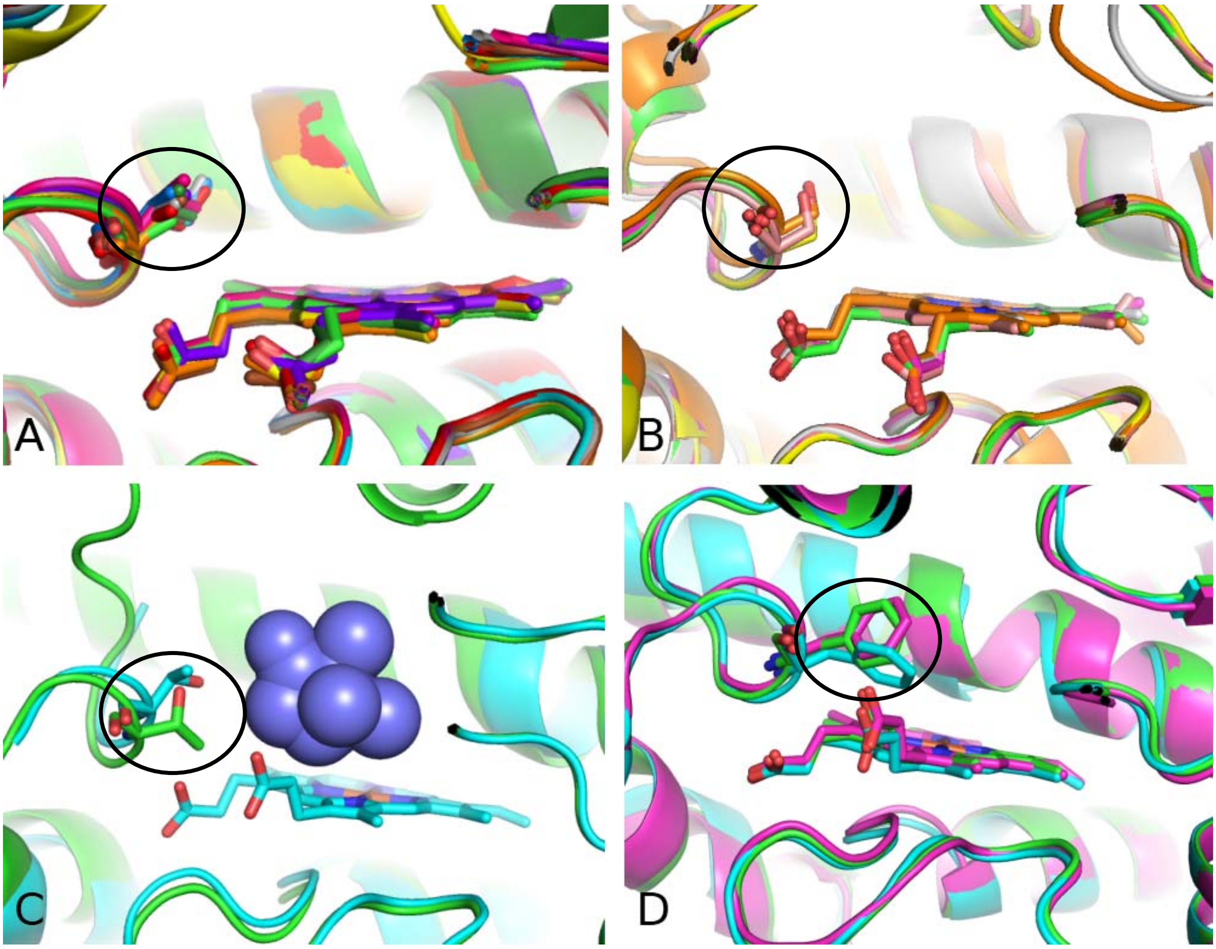

Figure S3 - Comparison of the F87 corresponding residues indicated (black circle) in multiple structures of the CYP2C family, CYP3A4, CYP101D ($P450_{cam}$) and CYP102A1 (P450 BM-3)

(A) Structures of the homologous family CYP2C were superimposed. A113 in CYP2C5 [PDBs: 1DT6 (light green), 1N6B (orange), 1NR6 (yellow)], I113 in CYP2C8 [PDBs: 2VN0 (cyan), 1PQ2 (brown), 2NNH (red), 2NNI (grey), 2NNJ (blue)] and V113 in CYP2C2 [PDBs: 1OG2 (dark green), 1OG5 (purple), 1R9O (magenta)] point in the direction of the heme. **(B)** Multiple structures of CYP3A4 were superimposed. For S119 it could be shown for all structures that it points towards the heme [PDBs: 1TQN (light green), 1W0E (pink), 1W0F (magenta), 1W0G (pink), 1J0D (grey), 2V0M (orange)]. **(C)** Open, substrate-free [PDB: 3L61] and closed, substrate-bound [PDB: 2CPP] CYP101D ($P450_{cam}$) both have T101 pointing towards the heme. Bound camphor is shown in blue. **(D)** F87 of CYP102A1 in points towards the heme in multiple structures [PDBs: 1BU7 (light green), 1FAG (cyan), 1BVY (magenta)].
